# The INVENT COVID trial: a structured protocol for a randomized controlled trial investigating the efficacy and safety of intravenous imatinib mesylate (Impentri®) in subjects with acute respiratory distress syndrome induced by COVID-19

**DOI:** 10.1186/s13063-022-06055-9

**Published:** 2022-02-16

**Authors:** Leila Atmowihardjo, Job R. Schippers, Imke H. Bartelink, Pierre M. Bet, Nienke van Rein, Keith Purdy, David Cavalla, Valérie Comberiati, Andrew McElroy, Sue D. Snape, Harm Jan Bogaard, Leo Heunks, Nicole Juffermans, Marcus Schultz, Pieter R. Tuinman, Lieuwe D. J. Bos, Jurjan Aman

**Affiliations:** 1 Dept. of Intensive Care, Amsterdam UMC location AMC, Amsterdam, The Netherlands; 2 Dept. of Pulmonology, Amsterdam UMC location VUMC, Amsterdam, The Netherlands; 3 Hospital Pharmacy, Amsterdam UMC location VUMC, Amsterdam, The Netherlands; 4 Exvastat, Cambridge, England; 5 Simbec-Orion, Merthyr Tydfil, England; 6 Dept. of Intensive Care, Amsterdam UMC, location VUMC, Amsterdam, The Netherlands; 7 Dept. of Intensive Care, Onze Lieve Vrouwe Gasthuis (OLVG), Amsterdam, The Netherlands

**Keywords:** COVID-19, Randomized controlled trial, Protocol, ARDS, Imatinib, Extravascular lung water, Vascular permeability, Endothelial dysfunction

## Abstract

**Background:**

The coronavirus disease 2019 (COVID-19) pandemic has led to a disruptive increase in the number of intensive care unit (ICU) admissions with acute respiratory distress syndrome (ARDS). ARDS is a severe, life-threatening medical condition characterized by widespread inflammation and vascular leak in the lungs. Although there is no proven therapy to reduce pulmonary vascular leak in ARDS, recent studies demonstrated that the tyrosine kinase inhibitor imatinib reinforces the endothelial barrier and prevents vascular leak in inflammatory conditions, while leaving the immune response intact.

**Methods:**

This is a randomized, double-blind, parallel-group, placebo-controlled, multicenter clinical trial of intravenous (IV) imatinib mesylate in 90 mechanically ventilated subjects with COVID-19-induced ARDS. Subjects are 18 years or older, admitted to the ICU for mechanical ventilation, meeting the Berlin criteria for moderate-severe ARDS with a positive polymerase chain reaction test for SARS-CoV2. Participants will be randomized in a 1:1 ratio to either imatinib (as mesylate) 200 mg bis in die (b.i.d.) or placebo IV infusion for 7 days, or until ICU discharge or death. The primary study outcome is the change in Extravascular Lung Water Index (EVLWi) between day 1 and day 4. Secondary outcome parameters include changes in oxygenation and ventilation parameters, duration of invasive mechanical ventilation, number of ventilator-free days during the 28-day study period, length of ICU stay, and mortality during 28 days after randomization. Additional secondary parameters include safety, tolerability, and pharmacokinetics.

**Discussion:**

The current study aims to investigate the efficacy and safety of IV imatinib in mechanically ventilated subjects with COVID-19-related ARDS. We hypothesize that imatinib decreases pulmonary edema, as measured by extravascular lung water using a PiCCO catheter. The reduction in pulmonary edema may reverse hypoxemic respiratory failure and hasten recovery. As pulmonary edema is an important contributor to ARDS, we further hypothesize that imatinib reduces disease severity, reflected by a reduction in 28-day mortality, duration of mechanical ventilation, and ICU length of stay.

**Trial status:**

Protocol version and date: V3.1, 16 April 2021. Recruitment started on 09 March 2021. Estimated recruitment period of approximately 40 weeks.

**Trial registration:**

ClinicalTrials.govNCT04794088. Registered on 11 March 2021.

## Administrative information

Note: the numbers in curly brackets in this protocol refer to SPIRIT checklist item numbers. The order of the items has been modified to group similar items (see http://www.equator-network.org/reporting-guidelines/spirit-2013-statement-defining-standard-protocol-items-for-clinical-trials/).

**Table:** 

Title {1}	A randomized, double-blind, placebo-controlled study to investigate the safety and efficacy of intravenous imatinib mesylate (Impentri®) in subjects with Acute Respiratory Distress Syndrome induced by COVID-19 (INVENT COVID)
Trial registration {2a and 2b}.	The trial was registered on ClinicalTrials.gov on 11-03-2021. The identification number is NCT04794088. The trial is also registered on the EudraCT database. The EudraCT number is 2020-005447-23
Protocol version {3}	Version 3.1, 16 April 2021
Funding {4}	European Union
Author details {5a}	Dr. J. Aman, Dept. of Pulmonary DiseasesAmsterdam UMC, Amsterdam, The Netherlands L. Atmowihardjo, Dept. of Intensive CareAmsterdam UMC, Amsterdam, The Netherlands Dr. L.D.J. Bos, Dept. of Pulmonary Diseases & Dept. of Intensive CareAmsterdam UMC, Amsterdam, The Netherlands J.R. Schippers, Dept. of Pulmonary DiseasesAmsterdam UMC, Amsterdam, The Netherlands
Name and contact information for the trial sponsor {5b}	Dr. J. Aman, Dept. of Pulmonary DiseasesAmsterdam UMC, Amsterdam, The Netherlands. E-mail: j.aman@amsterdamumc.nl
Role of sponsor {5c}	This is an investigator-initiated study. The sponsor is responsible for funding, study design, management, analyses, interpretation of data and publishing the data.

## Introduction

### Background and rationale {6a}

#### Background of ARDS due to COVID-19

The coronavirus disease 2019 (COVID-19) pandemic has led to an unanticipated increase in the number of patients with acute respiratory distress syndrome (ARDS) admitted to the intensive care unit (ICU), contributing to high morbidity and mortality. In these severe cases, COVID-19 infection is characterized by damage to the alveolocapillary wall and extensive pulmonary capillary leakage. The resulting alveolar flooding leads to impairment of oxygen diffusion and severe hypoxemic respiratory failure. In total, 17–42% of all hospitalized COVID-19 patients develop ARDS, requiring ICU admission or even invasive mechanical ventilation (29–91%). According to recent reviews, mortality may mount up to 15–20% (hospitalized patients) to even 40% in ICU patients [42]. This makes mortality in ICU-treated COVID-19 patients comparable to mortality in ARDS patients [13].

ARDS is characterized by acute onset, bilateral infiltrates on chest imaging due to pulmonary edema and severe hypoxemia despite mechanical ventilation. ARDS results from an overwhelming inflammatory process in the lung, involving damage to the alveolar epithelial and vascular endothelial cells. The early phase of ARDS is characterized by alveolar flooding with protein-rich fluid due to increased vascular permeability. Pulmonary edema in turn leads to poor lung compliance, bilateral infiltrates, and severe hypoxemia [25]. Recently implemented guidelines recommend dexamethasone [34] and tocilizumab [14] to modulate the inflammatory response in COVID-19. However, there is no registered drug to target the increased pulmonary vascular permeability which is seen in ARDS [26].

#### Rationale for intervention

Previous studies have shown that the anti-leukemic drug imatinib effectively and consistently protects against pulmonary vascular leak and alveolar edema during inflammatory stimuli [3]. It was shown that imatinib exerts its protective effect by inhibiting the non-receptor tyrosine kinase Arg/Abl2, a kinase involved in cytoskeletal remodeling in the endothelium during inflammation [2, 3]. The protective effects of imatinib were confirmed by several studies by independent research groups. These effects are reviewed elsewhere [36]; a summary is provided in Table 1. The protective effects of imatinib on endothelial barrier integrity were found at plasma concentrations of 2–10 μM [3], which is comparable to plasma levels in patients treated with imatinib for chronic myeloid leukemia (2–5 μM) [39]. This indicates that regular dosing schemes of imatinib are sufficient to protect the endothelial barrier.

**Table:** Table 1

Model	Effect of imatinib	Reference
***In vitro*** **studies**		
Rat aortic endothelial cells	Protects endothelial barrier	[20]
Human umbilical vein endothelial cells	Protects endothelial barrier Improves cell-matrix adhesion	[3]
Human lung microvascular endothelial cells	Protects endothelial barrier	[3]
Immortalized endothelial cells	Protects endothelial barrier	[9]
Human umbilical vein endothelial cells	Protects endothelial barrier	[18]
Mouse lung microvascular endothelial cells	Protects endothelial barrier	[40]
***In vivo*** **studies**		
Bleomycin-induced lung injury	Anti-inflammatory Anti-fibrotic	[35]
Isolated perfused lung model (mouse)	Inhibits lung vascular leak	[3]
Miles assay (mouse)	Attenuates vascular leak in skin	[3]
Cecal Ligation & Puncture (Sepsis) (mouse)	Attenuates vascular leak in lungs, kidneys	[3]
Intratracheal LPS (mouse)	Attenuates pulmonary edema	[18]
Ischemia-reperfusion in reperfusion lung	Reduces endothelial cytotoxicity	[40]
Miles assay (mouse)	Attenuates vascular leak in skin	[9]
Intratracheal LPS (mouse)	Attenuates vascular leak and inflammation	[23]
Cardiac bypass surgery	Attenuates vascular leak, improves perfusion, improves oxygenation	[19]
**Clinical case reports (human)**		
Pulmonary veno-occlusive disease	Resolution of pulmonary edema Improvement of oxygenation	[30]
Bleomycin-induced pneumonitis / lung injury	Resolution of pulmonary edema	[8]
Idiopathic pulmonary vascular leak	Resolution of generalized edema Reduction of pulmonary vascular leak	Aman 2013
Drug-induced pneumonitis	Resolution of pneumonitis, case series	[22]

The COUNTER-COVID study, a randomized, placebo-controlled clinical trial, evaluated the effect of oral imatinib on the need for oxygen suppletion in patients hospitalized with COVID-19. Although no difference was observed in the time to liberation from oxygen suppletion, a reduction in mortality [HR 0.51] and a reduction in the duration of mechanical ventilation and ICU length of stay was observed [4], suggesting that vascular leak plays a role particularly in more severe forms of COVID-19 and that imatinib may exert its protective effect in this population. We therefore hypothesize that improved endothelial barrier function through the administration of imatinib during the exudative phase of ARDS will translate into a decrease in the Extravascular Lung Water Index (EVLWi), as measured by PiCCO (Pulse Contour Cardiac Output) monitoring.

Concerning safety, imatinib was shown to have little effect on the immune response. Although developed to target leukemic cells, a normal lymphocytic response was observed in lymphocytes from patients treated with imatinib [24]. Of particular relevance to the current protocol is the fact that treatment with imatinib does not affect the control of primary viral infections [28]. Finally, extensive safety data on imatinib in COVID-19 were reported in the COUNTER-COVID study, which did not reveal any imatinib-related safety concerns in the 197 COVID-19 patients treated with imatinib [4]. Gastrointestinal absorption is unreliable in critically ill patients, among others due to disturbed bowel motility and intestinal edema [16, 27]. For this reason, an intravenous (IV) formulation was developed to achieve stable drug levels in the study population. IV infusion of imatinib has been safely administered to healthy volunteers and showed absolute (> 98%) bioavailability [32].

In summary, the main aims of the study are twofold: firstly, to investigate the effect of IV imatinib on pulmonary edema and clinical outcomes in mechanically ventilated patients with COVID-19-induced ARDS. Secondly, we aim to evaluate of the safety of IV imatinib in this patient group.

### Objectives {7}

The study’s primary objective is to evaluate the effect of IV imatinib compared to placebo on limiting the development of EVLWi in invasively mechanically ventilated subjects with COVID-19-related ARDS. EVLWi is the amount of fluid that is located in the interstitial and alveolar spaces. This measurement is representative of pulmonary edema, which is the main cause of hypoxemic respiratory failure in ARDS.

The secondary objectives of the study are as follows: to evaluate the effect of intravenous imatinib compared to placebo on patient outcomes in mechanically ventilated subjects with COVID-19-related ARDS; to evaluate the safety and tolerability of intravenous imatinib compared to placebo in mechanically ventilated subjects with COVID-19-related ARDS; and to determine imatinib pharmacokinetics in subjects with COVID-19-related ARDS.

### Trial design {8}

This is a randomized, double-blind, parallel-group, placebo-controlled, multicenter clinical trial comparing the effects of intravenous imatinib with placebo in mechanically ventilated subjects with COVID-19-related ARDS. The study will enroll 90 subjects (45 subjects/treatment arm, see sample size calculation). In this two-arm study, eligible subjects will be randomly assigned to receive imatinib or placebo in a 1:1 ratio. All subjects will receive standard of care according to local treatment protocols. At each of the participating centers, standard treatment protocols may include COVID-19-specific medication (e.g., dexamethasone and tocilizumab), low tidal volume ventilation, conservative fluid management, and prone positioning in case of persistent low partial pressure of oxygen / fraction of inspired oxygen (PaO2/FiO2) ratio.

## Methods: participants, interventions and outcomes

### Study setting {9}

The research population will be recruited from subjects admitted to the ICU with moderate-severe COVID-19 ARDS, as defined by the Berlin criteria Table 2 [6]. Patient recruitment will take place in the mixed (medical and surgical) ICUs of the Amsterdam University Medical Center (AUMC), locations AMC and VUMC, Erasmus University Medical Center Rotterdam, OLVG (Onze Lieve Vrouwe Gasthuis) hospital in Amsterdam, and the Dijklander hospital. Additional locations can be considered to support recruitment. The trial received ethical approval from the medical ethics review committee of the Amsterdam UMC, location VUMC, on 22.01.2021 (file number 2020.0752).

**Table 2:** Berlin definition of acute respiratory distress syndrome

Timing	Within 1 week of a known clinical insult or new or worsening respiratory symptoms
Chest imaging^a^	Bilateral opacities—not fully explained by effusions, lobar/lung collapse, or nodules
Origin of edema	Respiratory failure not fully explained by cardiac failure or fluid overload.
	Need objective assessment (e.g., echocardiography) to exclude hydrostatic edema if no risk factors present
Oxygenation^b^	
Mild	200 mmHg < PaO_2_/FIO_2_ ≤ 300 mmHg with PEEP or CPAP ≥ 5 cmH_2_O^c^
Moderate	100 mmHg < PaO_2_/FIO_2_ ≤ 200 mmHg with PEEP ≥ 5 cmH_2_O
Severe	PaO_2_/FIO_2_ ≤ 100 mmHg with PEEP ≥ 5 cmH_2_O

Abbreviations: *CPAP*, continuous positive airway pressure; *FIO*_*2*_, fraction of inspired oxygen; *PaO*_*2*_, partial pressure of arterial oxygen; *PEEP*, positive end-expiratory pressure

^a^Chest radiograph or computed tomography scan

^b^If altitude is higher than 1000 m, the correction factor should be calculated as follows: [PaO_2_/FIO_2_ − (barometric pressure/760)]

^c^This may be delivered noninvasively in the mild acute respiratory distress syndrome group

Reproduced from: ARDS Definition Task Force, Ranieri VM, Rubenfeld GD, et al. Acute respiratory distress syndrome: the Berlin Definition. JAMA 2012;307:2526-33

### Eligibility criteria {10}

#### Inclusion criteria

To be eligible to participate in this study, a subject must meet all of the following criteria: age ≥ 18 years; moderate or severe ARDS, as defined by Berlin definition for ARDS, and intubated for mechanical ventilation; polymerase chain reaction test positive for SARS-CoV2 within the current disease episode; provision of signed written informed consent from the patient or patient’s legally authorized representative.

#### Exclusion criteria

A potential subject who meets any of the following criteria will be excluded from participation in this study:

- Persistent septic shock (> 24 h) with a mean arterial pressure ≤ 65 mmHg and serum lactate level > 4 mmol/L (36 mg/dL) despite adequate volume resuscitation and vasopressor use (norepinephrine > 0.2 μg/kg/min) for > 6 h;
- Pre-existing chronic pulmonary disease, including known diagnosis of interstitial lung disease; known diagnosis of chronic obstructive pulmonary disease GOLD Stage IV or forced expiratory volume in 1 s < 30% predicted; diffusing capacity for carbon monoxide < 45% (if test results are available); total lung capacity < 60% of predicted (if test results are available);
- Chronic home oxygen treatment;
- Pre-existing heart failure with known left ventricular ejection fraction < 40%;
- Active treatment of hematological or non-hematological cancer with targeted immuno- or chemotherapy, or thoracic radiotherapy in the last year;
- Currently receiving extracorporeal life support;
- Severe chronic liver disease with Child-Pugh score > 12;
- Subjects in whom a decision to withdraw medical care is made (e.g., palliative setting);
- Inability of the ICU staff to initiate investigational medicinal product administration within 48 h of intubation;
- Known to be pregnant or breast-feeding;
- Enrolled in a concomitant clinical trial of an investigational medicinal product;
- White blood cell count < 2.5 × 10^9^/l; hemoglobin < 4.0 mmol/l or thrombocytes < 50 × 10^9^/l;
- The use of strong CYP3A4 inducers, including the following drugs: Carbamazepine, efavirenz, enzalutamide, phenobarbital, phenytoin, hypericum (St. John’s wort), mitotane, nevirapine, primidone, rifabutin, rifampicin;
- The presence of an intra-aortic balloon pump (IABP);
- Known medical history of aortic aneurysm in the trajectory of the PiCCO measurement between central venous line and arterial detector;
- Known medical history of an intracardiac shunt.

### Who will take informed consent? {26a}

Informed consent will be obtained by a good clinical practice (GCP) and protocol-trained investigator on site.

The recruitment of study subjects is as follows:

If a legal representative is present/accessible upon ICU admission:

The treating physician asks the legal representative if they would consider their relative participating in a clinical trial for which their relative may be eligible and obtains their permission for a member of the study team to contact the legal representative.

If the legal representative is not present/accessible at ICU admission:

The treating physician contacts the study team who initiates the deferred consent procedure. During the deferred consent period, a member of the study team will regularly attempt to contact the patient’s legal representative to obtain consent. In case that the legal representative cannot come to the hospital within the deferred consent period (e.g. due to COVID-19-related quarantine), the consent procedure will be done by phone and e-mail. The legal representative will first be informed by phone. After the phone call, the legal representative will receive the participation information including the informed consent form by e-mail, after which the reflection period of at least 6 h begins. To confirm participation, the legal representative will sign the informed consent form and send a copy by e-mail. The investigator will sign the copy and return the completed informed consent form. A signed hardcopy will be returned to the investigator personally, or by mail. The investigator will in turn send a double signed informed consent form to the legal representative.

#### Informed consent

The investigator gives the patient information and consent statement to the patient’s legal representative, answering any questions about the study they may have and provide additional information when requested. The legal representative has at least 6 h to read and consider whether to provide consent. The informed consent form is signed within 48 h from ICU admission.

## Interventions

### Explanation for the choice of comparators {6b}

The study is a placebo-controlled trial. The placebo consists of an isotonic sterile solution buffered with 0.01 M acetate (adjusted to pH 5.0) and the tonicity adjustment is by use of glycerol (1.9% v/v). A placebo has been chosen because it will not give any beneficial effect and thus the placebo group is a good representation of the standard of care.

### Intervention description {11a}

The intervention is IV imatinib. The infusion consists of an isotonic sterile solution of imatinib (Impentri®) 8 mg/ml, equivalent to 9.557 mg/mL of imatinib mesylate, buffered with 0.01 M acetate (adjusted to pH 5.0), and the tonicity adjustment is by use of glycerol (1.9% v/v).

A 25 ml volume of the investigational medicinal product (IMP) will be administered over 2 h as an IV infusion. This corresponds to a dose of 200 mg imatinib (100 mg/h, 12.5 ml/h), or 25 ml placebo (12.5 ml/h). Treatment will be administered twice daily (400 mg total daily imatinib dose) for up to 7 days, or until ICU discharge or death. The first dose of the IMP will be administered on day 1, as soon as possible after randomization. IMP should be administered 12 h (± 2 h) apart between 06:00 and 10:00 in the morning and 18:00 and 22:00 in the evening. On day 1, the first dose of IMP may be administered between 04:00 and 12:00 (08:00 ± 4 h) and the second dose between 16:00 and 24:00 (20:00 ± 4 h). Patients commencing treatment after 12:00 on day 1 should receive only one dose of IMP (between 16:00 and 24:00).

The administration should be by a central venous catheter, which is part of standard patient care. During the administration of the IMP, concomitant medication should not be given via the same central venous catheter but via other indwelling venous catheters.

Full details on the formulations are enclosed in the Investigational Medicinal Product Dossier (IMPD). The investigator’s brochure and IMPD will be available to the site investigator and will also be kept on file in the trial master file (TMF).

### Criteria for discontinuing or modifying allocated interventions {11b}

Participants have to stop with the IMP if they meet any of these criteria: leukocytes < 2.0 × 10^9^/L; thrombocytes < 50 × 10^9^/L, aspartate transaminase (AST) / alanine transaminase (ALT): elevation of > 10× upper limit of normal (ULN) in case of AST/ALT within reference values at baseline/inclusion or an elevation of > 10× baseline in case of elevated AST/ALT at baseline/inclusion; bilirubin: elevation of > 3× ULN in case of bilirubin levels within reference values at baseline/inclusion or an elevation of > 3× baseline in case of elevated bilirubin levels at baseline/inclusion; occurrence of life-threatening arrhythmias, including Torsade-de-Pointe, ventricular fibrillation, or ventricular tachycardia.

### Strategies to improve adherence to interventions {11c}

To improve adherence to the intervention protocol, standard operating procedures (SOPs) have been written and all investigators have been trained to adhere to the SOPs. The topics of these SOPs include screening and study inclusion, blood sampling, PiCCO measurements, and lung ultrasound.

### Relevant concomitant care permitted or prohibited during the trial {11d}

#### Concomitant medication

Subjects included in the study will be otherwise treated by the ICU staff not involved in the study, according to institutional and international guidelines. Throughout the study investigators/treating physicians may prescribe any concomitant medications or treatments deemed necessary to provide adequate supportive care. Concomitant medications prescribed for the patient will be reviewed daily while the patient is in the ICU. Use of anti-coagulants, systemic steroids, drugs that inhibit CYP3A4, antibiotics, tocilizumab, or other clinically relevant medications (e.g. vasopressors or drugs used to treat adverse events) will be recorded daily in the electronic Case Report Form (eCRF) between day 1 and day 10.

#### Concomitant supportive care

Supportive measures for ARDS include lung-protective ventilation [1] and, where indicated, ventilation in prone positioning [15], treatment with neuromuscular blockers [31], extracorporeal membrane oxygenation (ECMO) [10, 37], and restrictive fluid management [Wiedemann 2006], may be applied at the treating physicians’ discretion. Application of prone positioning, neuromuscular blockers, or ECMO should be recorded in the eCRF, together with the duration of the procedure in hours. Directions for concomitant supportive care include:

#### Ventilator settings

Ventilation setting should be in accordance with existing guidelines for subjects with ARDS. This consists of low tidal volumes targeting ± 6 ml/kg ideal body weight and titrated positive end-expiratory pressure (PEEP) levels. Also, low driving pressures could be considered.

#### Oxygenation targets

The oxygenation target ranges for SpO_2_ and PaO_2_ are 92 to 96%, and 8 to 11.5 kPa, respectively.

#### Sedation

Sedation follows the local guidelines for sedation in each participating unit. In general, these guidelines favor the use of analgo-sedation over hypno-sedation, the use of bolus over continuous infusion of sedating agents, and the use of sedation scores.

#### Fluid balance

A fluid balance targeted at normovolemia and diuresis of ≥ 0.5 ml/kg/h should be maintained, with low-threshold use of furosemide to maintain normovolemia. Crystalloid infusions are preferred over colloid infusions.

### Provisions for post-trial care {30}

The sponsor/investigator has liability insurance which is in accordance with article 7 of the Dutch Medical Research Involving Human Subjects Act (WMO).

The sponsor also has insurance that is in accordance with the legal requirements in the Netherlands (Article 7 WMO). This insurance provides cover for damage to research subjects through injury or death caused by the study. The insurance applies to the damage that becomes apparent during the study or within 4 years after the end of the study.

### Outcomes {12}

#### Main study outcomes

The main study outcome is the change in Extravascular Lung Water Index (EVLWi) between baseline (day 1) and day 4. The primary endpoint will be expressed as mean ± standard deviation. This parameter was chosen as it is a direct measurement of pulmonary edema.

#### Secondary study outcomes

The secondary study outcomes include the effect of intravenous imatinib compared to placebo on clinical outcomes (i.e. ventilator parameters and organ function) in mechanically ventilated subjects with COVID-19-related ARDS, the evaluation of the safety and tolerability of intravenous imatinib compared to standard of care, and the pharmacokinetics of intravenous imatinib.

#### Ventilator parameters

The difference and change from baseline in ventilator parameters, oxygenation, and lung mechanics between patients receiving imatinib versus the standard of care will be investigated. The following parameters will be used:

- Pulmonary vascular permeability index (days 1, 2, 4 and 7), expressed as PVPi = EVLW / PBV (pulmonary blood volume), where PBV = ITBV (intra-thoracic blood volume) − GEDV (global-end diastolic volume)
- Oxygenation index (days 1, 2, 4, 7, 10 and day 28, if available), expressed as OI = mean airway pressure × FiO_2_ × 100 / PaO_2_ (in kPa)
- PaO_2_/FiO_2_ ratio (days 1, 2, 4, 7, 10 and day 28, if available)
- Airway driving pressure (days 1, 2, 4, 7, 10 and day 28, if available)
- Compliance of the respiratory system (days 1, 2, 4, 7, 10 and day 28, if available)
- Mechanical power (days 1, 2, 4, 7, and 10 and day 28, if available), expressed as MP = 0.098 × RR × *V*_*T*_ × [PEEP + ΔP_insp_]

#### Organ function and outcome

The difference and change in clinical outcome will be investigated by recording the following:

- Sequential Organ Failure Assessment (SOFA) score (days 1, 2, 4, 7, 10 and day 28, if available);
- Number of ventilator-free days and alive at day 28;
- Duration of mechanical ventilation (days) between days 1 and 28;
- Length of ICU stay (days) between days 1 and 28;
- Hospital length of stay (days) between days 1 and 28;
- 28-day mortality.
- 9-point WHO ordinal Scale for Clinical Improvement [43]

#### Safety parameters

The following parameters will be recorded to evaluate drug safety:

- Blood cell count, i.e. hemoglobin, thrombocytes and leucocytes (days 1, 2, 4, 7 and 10);
- Kidney function, estimated glomerular filtration rate, sodium, and potassium (days 1, 2, 4, 7 and 10);
- Liver enzymes, i.e., AST, ALT, alkaline phosphatase, γ-glutamyl transferase, total bilirubin (days 1, 2, 4, 7 and 10);
- N-terminal prohormone of brain natriuretic peptide (days 1, 2, 4, 7 and 10);
- Serious adverse events (SAE)/adverse events (AE);
- Corrected QT interval (days 1, 2, 4, 7 and 10).

#### Pharmacokinetics

Imatinib plasma levels will be measured throughout days 1 to 7. In post hoc analyses, imatinib pharmacokinetics will be analyzed, using the following parameters:

- Imatinib plasma levels on day 1, drawn during IMP infusion, approximately 2 h after the start of the infusion (i.e., at the end of the infusion) and at 4 and 8 h after the start of IMP infusion. Imatinib plasma levels once daily on days 2, 4, and 7; the time of each administration and blood sample will be recorded.
- Albumin, alpha-1-acid glycoprotein (AAG) on day 1 (at 4 and 8 h after the start of IMP infusion), and on days 2, 4 and 7.

### Participant timeline {13}

Participant timeline is shown in Table 3.

**Table 3:** Participant timeline

Study period	Screening	Baseline	Treatment							Follow-up	
Study day (± window)	0-1	1	2	3	4	5	6	7	10	11-27	28 (±3)
ELIGIBILITY											
(Deferred) Informed consent	X										
Demographics	X										
Relevant medical history	X										
SARS-CoV-2 diagnostic test review	X										
Assess ARDS diagnosis and severity	X										
Inclusion and exclusion criteria	X										
STUDY INTERVENTION											
Randomization		X									
IMP administration		X	X	X	X	X	X	X			
Treatment with SoC	X	X	X	X	X	X	X	X	X	X	X
STUDY PROCEDURES											
12-lead Electrocardiogram	X	X	X		X			X	X		
Height and weight		X							X		
Targeted physical examination		X	X	X	X	X	X	X			
Vital signs: temperature, pulse rate, blood pressure, respiratory rate, SpO_2_ and FiO_2_	X	X	X	X	X	X	X	X	X		
Placement of central venous catheter and PiCCO catheter	X										
EVLWi and PVPi		X	X		X			X			
Mechanical ventilation parameters^(i)^		X	X		X			X	X		X
Arterial blood gas		X	X		X			X	X		
SOFA score and OI		X	X		X			X	X		X
Clinical status		X	X	X	X	X	X	X	X		X
PK sampling		X	X^(m)^		X^(m)^			X^(m)^			
Blood, plasma and serum sampling for biomarker studies		X	X		X			X	X		
Thoracic ultrasound (optional)		X			X			X			
Targeted medication review (including use of vasopressors)		X	X	X	X	X	X	X		X	X
Adverse event evaluation		X	X	X	X	X	X	X	X	X	X
SAFETY LABORATORY											
Hematology, chemistry, liver function tests, coagulation	X	X	X		X			X	X		
Pregnancy test for females of childbearing potential	X										

*EVLWi* Extravascular Lung Water Index; *FiO*_*2*_ fraction of inspired oxygen; *PaO*_*2*_ partial pressure of oxygen; *SARS-CoV-2* severe acute respiratory syndrome coronavirus 2; *SoC* standard of care; *SpO*_*2*_ peripheral oxygen saturation; *OI* Oxygenation Index; *PVPi* pulmonary vascular permeability index; *SOFA score* Sequential Organ Failure Assessment score

- To be performed prior to randomization.
- Or until hospital discharge, if earlier.
- If the patient has been discharged before day 28, this assessment may be conducted by telephone or with a home visit by study staff. For visits conducted by telephone, it will not be possible to perform some scheduled assessments (e.g., ECG). Where patients have discontinued the study prematurely, day 28 assessments should be performed, where possible.
- Medical history includes an estimate of date and time of first signs and symptoms and presence of co-morbidities (e.g., respiratory, cardiovascular, metabolic, malignancy, endocrine, gastrointestinal, immunologic, renal).
- Tests performed prior to hospital admission are acceptable provided the test result is from a laboratory or validated point of care test.
- Documentation of evidence to confirm diagnosis and ARDS severity according to Berlin definition.
- Randomization and the first dose of IMP must take place within 48 h of intubation. IMP should be administered twice daily, 12 h (± 2 h) apart between 06:00 and 10:00 in the morning and 18:00 and 22:00 in the evening. Exceptions apply on day 1—see Intervention description {11a}
- Where clinically indicated.
- Air driving pressure; respiratory system compliance, PEEP, tidal volume, and mechanical power.
- OI: Assess once daily, in case of more measurements per calendar day, the worst OI, and all related measurements will be taken. Manual techniques should be used only if automated devices are not available.
- Record any change in clinical status: change in extubation or reintubation; first unassisted breathing or death; discharge from ICU, hospital, or death; WHO ordinal Scale for Clinical Improvement.
- Plasma samples for the determination of imatinib, albumin, and AAG are taken at 4 h and between 7 and 8 h after the start of the first IMP infusion. Where PK sampling cannot be performed on day 1, samples will be taken 3 h and 7 h after the start of the first IMP infusion on day 2 instead. For a subgroup of patients, extra samples will be taken during the IMP infusion and 2 h after the start of the first infusion (i.e., at the end of the infusion).
- Pre-IMP infusion (am or pm dose).
- Laboratory tests performed in the 48 h prior to the first dose of study treatment will be accepted for determination of eligibility. If multiple tests are performed during this time, the test closest to dosing will be regarded as the formal sample to confirm eligibility.
- Any laboratory tests performed as part of routine clinical care within ± 1 day of day 10 assessment while hospitalized can be used.

### Sample size {14}

The number of subjects scheduled for inclusion in the study is 90, including 45 subjects in the placebo arm and 45 subjects in the imatinib mesylate arm.

Sample size calculations were done with the formula: *zα*/2 − *zπ* = (*n*/2)1/2 × (*μ*1 − *μ*2)/σ, in which *zα*/2 − *zπ* = 2,8 (*α* = 5% en *π* = 80%). The change (*Δ*) in EVLWi between day 0 and day 4 is the primary outcome. We expect the baseline EVLWi to be around 17 ml/kg, as previously described for patients with moderate-severe ARDS [17, 21]. In the placebo group, we expect the ΔEVLW day 0 and day 4 to be 0.5 (μ1), based on previous literature [11, 33]; in the imatinib group, we expect the ΔEVLWi to be − 4 (*μ*2). This is considered a clinically relevant difference as this difference was found to independently predict ARDS mortality in another clinical study [7]. The expected treatment effect is based on the imatinib effect observed in preclinical data: 25% reduction in vascular leak [3]. The sigma (*σ*) for ΔEVLWi is set at 7.0, based on previous EVLWi studies [11, 17]. Using the equation above, this yields 76 subjects with 38 subjects/arm. Taking into account a dropout rate of 15%, a total of 90 subjects will be recruited.

### Recruitment {15}

Subjects will be recruited in the ICUs of the AUMC, locations AMC and VUMC, the Onze Lieve Vrouwe Gasthuis (Amsterdam), the Erasmus Medical Center (Rotterdam), and the Dijklander hospital. Additional centers are under consideration. With a minimum of 5 active centers and an anticipated duration of the trial of 18 months, and a target number of inclusions of 90 subjects, this means that 90/5 = 18 subjects per center need to be enrolled, which amounts to < 0.25 subjects/center/week. When considering the AMC and VUMC as representative hospitals, the required rate of inclusion/center is below the current rate of presentations/center.

## Assignment of interventions: allocation

### Sequence generation {16a}

Subjects will be randomized 1:1 to receive a placebo or imatinib. Randomization will take place via Castor Electronic Data Capture (EDC) using a computer-generated allocation sequence based on variable block sizes (2–6 patients/block) with stratification per participating center.

### Concealment mechanism {16b}

Upon randomization in Castor EDC, the pharmacy receives an automatic notification of the study participant’s allocation, which is not visible to the investigators. Subsequently, an order for the IMP is placed in the electronic patient record, which directly informs the pharmacy to start preparation of the IMP.

### Implementation {16c}

The researcher who includes the participant fills in the eligibility criteria in the eCRF and then randomizes the participant using the ‘Randomize’ button in Castor EDC. The researcher specified the method of computer-generated allocation sequence generation by variable block sizes in Castor EDC before the start of the study. The automatic allocation sequence determines the assignment of the patient to the blinded intervention. After randomization, the patient is enrolled into the study.

## Assignment of interventions: Blinding

### Who will be blinded {17a}

This is a double-blind study. Subjects, clinical staff (nurses and physicians), and investigators will be blinded for study medication, to prevent reporting bias.

The imatinib solution is slightly yellow, while the placebo is transparent. Therefore, blinding will be guaranteed by using colored syringes and infusion lines containing the IMP, which ensures that the content will not be visible during treatment. Investigators will remain blinded to each participant’s assigned study treatment throughout the course of the study. To maintain this blind, an otherwise uninvolved third party will be responsible for the reconstitution and dispensation of all study treatments and will endeavor to ensure that there are no differences in time taken to dispense following randomization.

### Procedure for unblinding if needed {17b}

Codebreaking is allowed in any of the following circumstances: treatment of an individual in a medical emergency where knowledge of the treatment allocation is required; in the event of a Suspected Unexpected Serious Adverse Reaction (SUSAR) the subject will be unblinded if this is required for treatment of the SUSAR; if the ICU staff/researcher is accidentally exposed to study medication.

The unblinding procedure will occur through the pharmacy.

## Data collection and management

### Plans for assessment and collection of outcomes {18a}

Data will be collected from the electronic patient record and manually entered in the eCRF. All investigators underwent Castor EDC training prior to the start of the study. All Castor users have assigned roles, which specify particular user rights. These roles include data entry, monitoring, data management, pharmacovigilance, and pharmacy.

### Plans to promote participant retention and complete follow-up {18b}

To prevent reallocation of patients to other centers, the intensive care physician in charge of patient transferal will be informed about inclusions by a daily e-mail and asked not to transfer study participants, if possible. To maintain the trust of the legal representative and promote further inquiry during the study if needed, the representative is provided with the contact details of the investigators and an independent expert.

Follow-up will be 28 days. Follow-up is ensured by keeping a subject identification log in which the date of ICU and/or hospital discharge is recorded. In case of hospital discharge, subjects are called on day 28 (± 3 days).

In case a patient or his/her relatives decide to stop the study medication (e.g., due to side effects / non-tolerance), the patient will be asked to stay in the study. In case a patient decides to stay in the study, follow-up will continue according to the study protocol. In case a patient decides to leave the study, follow-up will be discontinued while all collected data are stored.

### Data management {19}

Data management will be provided by the clinical trial management service company Simbec-Orion and data quality is furthermore monitored by the local Clinical Research Unit. To ensure data quality, validation checks and dependencies were programmed into the eCRF. Examples of these checks include accepted ranges for laboratory results and vital parameters, date and time checks and automated warning messages upon events such as protocol deviations and adverse events. A data validation plan, written by Simbec-Orion in cooperation with the investigators, specifies the nature of each validation check and warning message per variable and the way of programming the check in the statistical analysis. Data entry will be done by a select group of blinded investigators trained in the eCRF and the study protocol.

### Confidentiality {27}

The data generated in this study will be encoded with a unique patient ID, not based on the patient’s initials and birth date. The code unique to each subject will be used to identify data and the key to the code will be available to the investigators. Furthermore, personal data will comply with the General Data Protection Regulation Study data will be stored for the required minimum period of 15 years.

Preservation and registration of human material are performed at the Pulmonary Hypertension biobank. By saving serum and blood plasma at − 80 °C, analyses will be performed on biomarkers of metabolism, inflammation, epithelial and endothelial injury, as well as transcriptomics, metabolomics and proteomics. Human material will be stored for 5 years after enrolment and follow-up of the last study subject.

#### Source data

Source documents are original documents, data, and records from which subjects’ eCRF data are obtained. These include, but are not limited to, hospital records (from which medical history and previous and concurrent medication may be summarized into the eCRF), clinical and office charts, laboratory and pharmacy records, diaries, microfiches, radiographs, and correspondence. eCRF entries will be considered source data if the eCRF is the site of the original recording (i.e. there is no other written or electronic record of data).

Several data fields in the eCRF are considered source documents, in particular, the following data fields: smoking; ECG; parts of the in- and exclusion criteria, e.g., chronic oxygen use and concomitant use of other medication.

Although the electronic patient file is considered the primary source document, the grading of the severity of (severe) adverse events is not always mentioned in the electronic patient file and is therefore subject to the interpretation of the study physician.

All documents will be stored safely in confidential conditions. On all study-specific documents, other than the signed consent, the subject will be referred to by the study Patient ID code, not by name.

The only trial staff as listed on the Delegation Log shall have access to trial documentation other than the regulatory requirements listed below.

### Plans for collection, laboratory evaluation and storage of biological specimens for genetic or molecular analysis in this trial/future use {33}

Blood samples will be collected on days 1, 2, 4, 7, and 10.

- 2 ethylenediaminetetraacetic acid tubes (of 5 mL) and 1 heparin-gel tube (of 5 mL) will be drawn on days 1, 2, 4, 7, and 10 for clinical safety laboratory measurements (1 tube each), and to serve as study material (1 tube each).
- 1 Paxgene tube (of 2.5 mL) will be drawn on days 1 and 4 to serve as study material.
- A lithium heparin tube (4 mL) will be drawn during IMP infusion, 2 h after the start of IMP infusion (i.e., at the end of the infusion), 4 h and 8 h after the start of IMP infusion on day 1 for pharmacokinetic measurements.
- A lithium heparin tube (4 mL), two citrate tubes (2.7 mL), and a serum gel tube (5 mL) will be drawn on days 1, 2, 4, 7, and 10 to serve as study material.
- Arterial blood gas on days 1, 2, 4, 7, and 10.

The serum and plasma samples (referred to as “study material”) will be used for analyses of pharmacokinetics, immune responses, inflammatory parameters and parameters of endothelial and epithelial injury. These analyses will be performed as post hoc analyses.

Statistical methods

### Statistical methods for primary and secondary outcomes {20a}

The primary endpoint is the change in extravascular lung water index (ΔEVLWi) between day 1 (baseline) and day 4. The primary endpoint will be expressed as mean ± standard deviation, or in case of non-normal distribution, as median ± interquartile (IQR) range, and tested for the statistical difference using a *t*-test or a Mann-Whitney *U* test in case of non-normal distribution. In addition, the ANCOVA analysis will be performed.

For all parameters below, the statistical comparison will be performed between the placebo and the imatinib group (Table 4).

**Table 4:** Parameters

Parameter	Presentation	Test
PVPi (days 1, 2, 4, and 7)	Mean ± SD	Linear mixed model
Oxygenation index (days 1, 2, 4, 7, 10, and 28)	Mean ± SD	Linear mixed model
PaO_2_/FiO_2_ ratio (days 1, 2, 4, 7, 10, and 28)	Mean ± SD	Linear mixed model
Airway driving pressure (days 1, 2, 4, 7, 10, and 28)	Mean ± SD	Linear mixed model
Compliance (days 1, 2, 4, 7, 10, and 28)	Mean ± SD	Linear mixed model
Mechanical power (days 1, 2, 4, 7, 10, and 28)	Mean ± SD	Linear mixed model
SOFA score (days 1, 2, 4, 7, 10, and 28)	Mean ± SD	Linear mixed model
Number of ventilator-free days (days 1 to 28)	Mean ± SD	T-test
Duration of mechanical ventilation (days) (days 1 to 28)	Mean ± SD	T-test
Length of ICU stay (days) (days 1 to 28)	Mean ± SD	T-test
Hospital length of stay (days) (days 1 to 28)	Mean ± SD	T-test
28-day mortality	Number (%)	Cox prop. Hazards
Plasma biomarkers (days 1, 2, 4, 7, and 10)	Median ± IQR	MWU / Linear mixed model
Blood cell counts (days 1, 2, 4, 7, and 10)		
RBC	Mean ± SD	Linear mixed model
WBC	Mean ± SD	Linear mixed model
Thrombocytes	Mean ± SD	Linear mixed model
Kidney function (days 1, 2, 4, 7, and 10)		
Creatinine	Mean ± SD	Linear mixed model
eGFR	Mean ± SD	Linear mixed model
Liver enzymes (days 1, 2, 4, 7, and 10)		
ALT	Mean ± SD	Linear mixed model
AST	Mean ± SD	Linear mixed model
Bilirubin	Mean ± SD	Linear mixed model
γ-glutamyl transferase	Mean ± SD	Linear mixed model
Alkaline phosphatase	Mean ± SD	Linear mixed model
NT-proBNP (days 1, 2, 4, 7, and 10)	Median ± IQR	Linear mixed model
SAEs / AE	Number (%)	Descriptive
ECG (ΔQTc time day 1 versus day 4)	Mean ± SD	T-test

*IQR* interquartile range, *MWU* Mann-Whitney *U*, *SD* standard deviation

### Interim analyses {21b}

There will be no interim analyses on efficacy. Interim analyses on safety will be performed in accordance with the Data Safety and Monitoring Board (DSMB) and will be performed. The DSMB gives advice based on safety reports. The decision to terminate the study lies with the trial coordinator.

### Methods for additional analyses (e.g., subgroup analyses) {20b}

#### Other study parameters

Plasma concentrations of imatinib at *C*_trough_ will be described by descriptive statistics, including mean, SD, minimum, maximum, and median. Nonlinear mixed effect modeling as implemented in the NONMEM program (Version 6, Globomax LLC, Hanover, MD, USA) will be used for data analysis. S-Plus (Version 6.2, Insightful Software, Seattle WA) and R (version 7.2) will be used to visualize the data.

Using a previously validated imatinib PK model, the population pharmacokinetics of imatinib will be characterized in terms of clearance, the volume of distribution, and parameters that describe protein binding/ free fraction and bioavailability. Between-patient and, if applicable, within-patient variability in these parameters will be estimated. The goodness-of-fit of the population PK models will be judged by the goodness-of-fit plots (created by using Pirana and Xpose (version 4.3.2, Niclas Jonsson and Mats Karlsson, Uppsala, Sweden)) as well as the precision of the parameter estimates and the magnitude of residual variability. In addition, the objective function value (OFV) will be used to statistically assess the goodness of fit; whether the addition of a parameter in the model statistically significantly improves the fit will be determined with the likelihood ratio test. A *p*-value below 0.05 in a chi-squared distribution with one degree of freedom will be considered statistically significant.

##### Covariate analysis

To explain pharmacokinetic between- and within-patient variability, covariates will be tested in a two-step approach using the data of all available Impentri trial patients (oral and IV). In the first step, all different covariates will be introduced to the structural model separately and tested for their significance and improvement of the fit. A *p*-value of < 0.05, determined with the likelihood ratio test (OFV drop of at least 3.8 units), will be considered statistically significant during this step of the analysis. In the second step, all covariates selected during the first step will be included in the model, yielding the intermediate model. A backward elimination procedure (multivariate analysis) will subsequently be used to develop the final model. A covariate will be retained in the model if exclusion results are a statistically significant worsening of the fit, again determined with the likelihood ratio test. A *p*-value < 0.01, corresponding to an OFV increase of at least 6.6 units will be applied for this purpose to correct for the multiple testing phenomenon. Covariates that will be considered include estimated glomerular filtration rate (eGFR), sex, age, body weight and length (body surface area, body mass index), comorbidity, concomitant medication, AAG and albumin levels. The resulting PK model or PK derivatives (AUC *C*_max_ or *C*_through_ free/total) will be used for further outcome analysis.

### Methods in analysis to handle protocol non-adherence and any statistical methods to handle missing data {20c}

An intention-to-treat analysis will be used, including all patients that underwent randomization.

### Plans to give access to the full protocol, participant level-data and statistical code {31c}

The full protocol will be available, as well as the statistical analysis plan. The study database will be available in anonymized form upon request by third, academic partners.

## Oversight and monitoring

### Composition of the coordinating center and trial steering committee {5d}

The coordinating center is the Amsterdam UMC, location VUMC. It is an investigator-initiated study which means the Amsterdam UMC is responsible for funders, study design, management, analyses, interpretation of data, and publishing the data.

The steering committee members are:

Dr. J. Aman, Project leader, Dept. of Pulmonary DiseasesAmsterdam UMC, Amsterdam, The Netherlands

Prof. Dr. H.J. Bogaard, Dept. of Pulmonary DiseasesAmsterdam UMC, Amsterdam, The Netherlands

Dr. L.D.J. Bos, Principal investigator AMC, Dept. of Pulmonary Diseases & Dept. of Intensive CareAmsterdam UMC, Amsterdam, The Netherlands

Prof. dr. L. Heunks, Principal investigator VUmc, Dept. of Intensive CareAmsterdam UMC, location VUMC, Amsterdam, The Netherlands

Dr. N. Juffermans, Principal investigator OLVG, Dept. of Intensive CareOLVG Oost & Amsterdam UMC, Amsterdam, The Netherlands

Prof. dr. M.J. Schultz, Dept. of Intensive CareAmsterdam UMC, location AMC, Amsterdam, The Netherlands

Prof. dr. P.R. Tuinman, Dept. of Intensive CareAmsterdam UMC, location VUMC, Amsterdam, The Netherlands

The daily activities are handled by L. Atmowihardjo and J.R. Schippers under the supervision of L. Bos and J. Aman. Meetings for this team are weekly.

### Composition of the data monitoring committee, its role and reporting structure {21a}

The DSMB is composed of three persons. They are chosen for their expertise in either clinical trials, ARDS, or IMP studies on the ICU. The responsibilities of the DSMB are to evaluate study progress, make recommendations, and assist in solving problems raised by the chief investigator and advise the chief investigator whether or not to continue with the study. They also inform the METC and board of directors about this advice. We declare that the DSMB is independent and has no conflict of interest.

Furthermore, an independent monitor provided by the clinical research department of the VUMC conducts remote monitoring and on-site visits at all participating sites. Monitoring visits are scheduled after the inclusion of the first 2–3 patients, repeated after randomization of the 15th patient and after the last subject visit at a particular site. After database lock, remote close-out is conducted through completion of a close-out checklist by the local PI or delegate.

### Adverse event reporting and harms {22}

#### Reporting of adverse events (AE) and serious adverse events (SAE)

Due to the nature of the disease, the incidence of AEs and SAEs, as well as the risk of death due to the underlying condition, is high (the hospital mortality in ventilated ICU patients is 21%). In clinical studies conducted in patients with ARDS, changes in vital parameters are frequent, including temporary changes in blood pressure and gas exchange parameters, as are deviations in laboratory values and ECG values.

#### AE reporting

Given the high incidence of adverse events inherent to the nature of the underlying condition (i.e., ARDS), given the safety of imatinib as observed in COVID-19 pneumonitis before (COUNTER-COVID study), and given the regular recording of vital signs and blood and ECG parameters as safety indicators in the eCRF, we propose not to report AEs in a standard fashion, except for the following events: pulmonary embolism, as detected on contrast-enhanced chest CT, not leading to circulatory or pulmonary instability, the occurrence of infections, requiring initiation of antibiotic therapy; non-life-threatening infusion reactions, including but not limited to skin rash.

#### SAE reporting

Given the high incidence of adverse events inherent to the nature of the underlying condition (i.e., ARDS), and given the safety of imatinib as observed in COVID-19 pneumonitis before (COUNTER-COVID study), we propose not to report all serious events as SAEs.

The following SAEs will be reported: death due to any cause; cardiopulmonary SAEs such as the need for extracorporeal membrane oxygenation, cardiac events like arrhythmias requiring CPR or medical resuscitation, thrombo-embolic events with life-threatening circulatory or pulmonary instability, spontaneous bleeding, requiring blood transfusion, or surgical intervention or myocardial infarction. Furthermore reported will be the following: the need for renal replacement therapy; liver failure (hepatic SOFA score > 4); hematologic SAEs such as thrombocytopenia (< 50^10^9^/L), diffuse intravascular coagulation, leukocytopenia (< 2^10^9^/L), anemia (hemoglobin < 4 mmol/L); intracranial bleeding or ischemic stroke and life-threatening infusion reactions, requiring intensification of existing intensive care treatment, including additional vasopressor, fluid support, corticosteroids, and antihistamines. Lastly, any unexpected serious event judged as an “untoward medical occurrence” will be reported as an SAE.

If the trial site personnel are unable to complete the electronic SAE form within 24 h after receiving information about the event, the initial reporting must be done on the paper SAE report and e-mailed to Simbec-Orion Pharmacovigilance (within 24 h of awareness) at pharmacovigilance@simbecorion.com. The trial site should report the event in the electronic SAE form of the eCRF as soon as possible.

A medically qualified person at the trial site identified on the delegation log with this responsibility must assess the SAE. The Principal Investigator or delegated sub-investigators are responsible for the SAE reporting procedures at the site during the trial and must always sign off on each SAE (regardless of whether reported using the electronic or paper form) even if another site staff has reported the event on behalf of the investigators.

The following events will not be reported as SAEs: fall in blood pressure, requiring fluid resuscitation or inotropic medication; respiratory failure, requiring intensification of mechanical ventilation, prone positioning, or bronchial intervention; a decrease in eGFR or urine output, not requiring renal replacement therapy

#### Reporting of suspected unexpected serious adverse reactions (SUSARs)

Simbec-Orion pharmacovigilance holds the responsibility of submissions to the Netherlands Competent Authority and Central Ethics committee (EC) and will report the following SUSARs to the METC: SUSARs that have arisen during this clinical trial that was assessed by the METC and SUSARs that have arisen in other clinical trials of the same sponsor and with the same medicinal product and that could have consequences for the safety of the subjects involved in this clinical trial that was assessed by the METC.

The sponsor will provide expedited reports for all SUSARs to the competent authorities in the other Member States, according to the requirements of the Member States.

The expedited reporting will occur not later than 15 calendar days after the sponsor has first knowledge of the adverse reactions. For fatal or life-threatening cases, the term will be maximal 7 calendar days for a preliminary report with another 8 calendar days for completion of the report.

### Frequency and plans for auditing trial conduct {23}

Not applicable.

### Plans for communicating important protocol amendments to relevant parties (e.g., trial participants, ethical committees) {25}

A “substantial amendment” is defined as an amendment to the terms of the METC application, or to the protocol or any other supporting documentation, that is likely to affect to a significant degree: the safety or physical or mental integrity of the subjects of the trial; the scientific value of the trial; the conduct or management of the trial; or the quality or safety of any intervention used in the trial.

All substantial amendments will be notified to the METC and the competent authority.

Non-substantial amendments will not be notified to the accredited METC and the competent authority but will be recorded and filed by the sponsor.

### Dissemination plans {31a}

The data generated by this study will be presented at international conferences and published in key journals. The publication will be in accordance with the basic principles of the Central Committee on Research Involving Human Subjects (CCMO) statement on publication policy (see https://www.ccmo.nl/publicaties/publicaties/2002/03/15/ccmo-notitie-publicatiebeleid).

## Discussion

In the quest for novel treatment options for COVID-19-induced ARDS, the tyrosine kinase inhibitor imatinib showed promising results in models of inflammatory injury to the pulmonary vasculature. It stabilizes the alveolar-capillary barrier and prevents vascular fluid leakage, resulting in the hypothesis that imatinib treatment may reverse the detrimental alveolar edema observed in ARDS and thereby hasten recovery from respiratory failure.

A major strength of the study is that the driving pathophysiological processes of ARDS, pulmonary vascular inflammation and leak, and the clinical outcomes related to these processes, are evaluated in parallel. The extravascular lung water index (EVLWi) was chosen as the primary endpoint to test the hypothesis that imatinib reduces pulmonary edema, with potentially beneficial effects on secondary outcomes like mortality, time on mechanical ventilation, and ventilation parameters. The trial will therefore provide valuable insights into the role of pulmonary vascular leak in ARDS and its clinical outcomes. Currently, there is conflicting evidence surrounding the prognostic value of EVLWi in ARDS patients [BALTI 2006, [41]], with no high-quality prospective studies yet showing a clear benefit of decreased EVLWi on mortality and other clinical parameters. This study therefore also aims to add to the body of evidence investigating the clinical significance of changes in EVLWi for ARDS prognosis. As an additional strength of this study, biomarkers of inflammation and endothelial injury are measured, providing information on the various components of ARDS pathophysiology, as well as pharmacokinetic measurements, which will be used to model drug-effect relationships. Lastly, the double-blinded character of the study is powerful, limiting performance bias.

A possible difficulty might be other ongoing intervention trials involving COVID-19 patients, which could limit inclusion rate, particularly in the academic centers. This potential problem was addressed by including a variety of peripheral and academic hospitals as participating sites. Participant dropout due to transfer of recruited patients to non-participating centers was accounted for in the sample size calculation. Additionally, at the time of the submission of this protocol, the incidence of COVID-19-related ICU admissions is decreasing in the Netherlands, which poses a risk to reaching the target sample size. To account for the declining inclusion rate, the expansion of the inclusion criteria to encompass non-COVID-19-related ARDS will be considered.

Given the worldwide prevalence of ARDS and specifically COVID-19-induced respiratory failure, finding effective treatment options for the critically ill is of utmost importance. Imatinib could provide a valuable addition to the currently still sparse treatment options for ARDS. The INVENT COVID trial is the first randomized controlled trial investigating the efficacy and safety of IV imatinib in COVID-19-induced ARDS. It will provide valuable insights into imatinib as a strategy to protect the alveolocapillary barrier, which, if proven effective, may extend beyond the current coronavirus pandemic to treat non-COVID-19 ARDS.

## Abbreviations

AAG: Alpha-1-acid glycoprotein
ALT: Alanine transaminase
ARDS: Acute respiratory distress syndrome
AST: Aspartate transaminase
CCMO: Central Committee on Research Involving Human Subjects; in Dutch Centrale Commissie Mensgebonden Onderzoek
COVID-19: Coronavirus disease caused by infection with severe acute respiratory syndrome coronavirus 2 (SARS-CoV-2)
DSMB: Data Safety Monitoring Board
eCRF: Electronic case report file
EDC: Electronic Data Capture
EU: European Union
EudraCT: European drug regulatory affairs Clinical Trials
EVLW: Extravascular lung water
EVLWi: Extravascular lung water indexed against predicted body weight or surface area
FiO_2_: Fraction of inspired oxygen
GCP: Good Clinical Practice
ICU: Intensive care unit
IV: Intravenous
IMP: Investigational Medicinal Product
IMPD: Investigational Medicinal Product Dossier
IQR: Interquartile range
METC: Medical research ethics committee (MREC); in Dutch: medisch-ethische toetsingscommissie (METC)
OFV: Objective function value
PaO_2_: Partial pressure of arterial oxygen
PiCCO: Pulse Contour Cardiac Output. Used to measure a patient’s hemodynamic status.
PVPi: Pulmonary vascular permeability indexed against predicted body weight or surface area
(S)AE: (Serious) adverse event
SOFA: Sequential organ failure assessment
SOP: Standard operating procedure
Sponsor: The Sponsor is the party that commissions the organization or performance of the research for example a pharmaceutical company, academic hospital, scientific organization, or investigator. A party that provides funding for a study but does not commit is not regarded as the sponsor but referred to as a subsidizing party.
SUSAR: Suspected Unexpected Serious Adverse Reaction
TMF: Trial Master File
ULN: Upper limit of normal
WMO: Medical Research Involving Human Subjects Act; in Dutch: Wet Medisch-wetenschappelijk Onderzoek met Mensen
